# Editorial: Advances in polymer-based biomaterials for tissue engineering and regenerative medicine

**DOI:** 10.3389/fbioe.2026.1866948

**Published:** 2026-07-13

**Authors:** Alina Kirillova, Chandrasekhar R. Kothapalli, Deepak M. Kalaskar, Linqing Li

**Affiliations:** 1 Department of Materials Science and Engineering, Iowa State University, Ames, IA, United States; 2 Department of Chemical and Biomedical Engineering, Cleveland State University, Cleveland, OH, United States; 3 Department of Surgery and Intervention Science, University College London, London, United Kingdom; 4 Department of Chemical Engineering and Bioengineering, University of New Hampshire, Durham, NH, United States

**Keywords:** 3D printing, bioactive materials, biomimetic scaffolds, hydrogel systems, polymer-based biomaterials, regenerative medicine, structure-property relationships, tissue engineering

## 1 Introduction and scope

**FIGURE 1:**
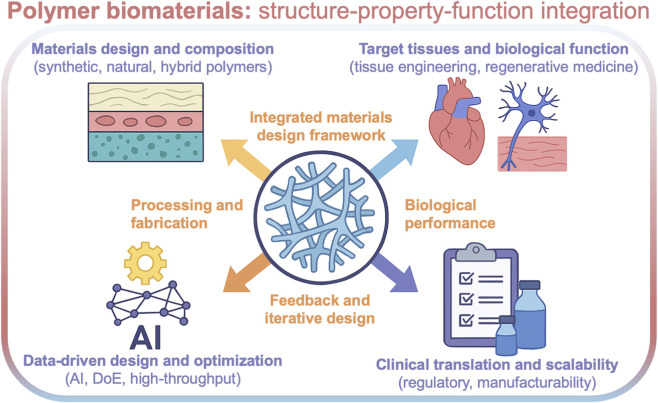
Integrated design framework for polymer-based biomaterials in tissue engineering and regenerative medicine. Materials composition and processing determine structure-property-function relationships that govern biological performance in target tissues and inform clinical translation. Data-driven approaches enable iterative feedback and optimization, establishing a closed-loop system for the development of multifunctional biomaterials with improved performance and scalability.

Polymer-based biomaterials have become a central platform in tissue engineering and regenerative medicine due to their versatility, tunability, and compatibility with advanced fabrication techniques (Langer and Vacanti, 1993; Place et al., 2009; Murphy and Atala, 2014). Recent advances increasingly focus on designing materials that not only provide structural support but also actively regulate cellular behavior through controlled mechanical, biochemical, and microenvironmental cues (Discher et al., 2005; Wang et al., 2020). This Research Topic brings together original research and review articles that collectively highlight emerging strategies in the design, processing, and functional integration of polymer-based biomaterials across a range of tissue applications. Particular emphasis is placed on linking material chemistry, processing routes, and multiscale architecture to biological function and translational potential (Figure 1).

## 2 Synthesis of contributions and emerging themes

The **original research articles** in this Research Topic highlight advances in polymer-based biomaterials for bone, tendon, neural, dermal, and cardiac tissue engineering, with a strong focus on tunable structure-property-function relationships.

Anup et al. explore silk fibroin scaffolds produced by electrospinning and cryogelation as complementary platforms for modeling the bone-tendon interface. Electrospun scaffolds provide aligned fibrous structures and higher stiffness suitable for tendon-like regions, while cryogels produce porous, compliant matrices that promote cell infiltration on the bone side. The combined multiphase system demonstrates how spatially distinct mechanical and structural cues can support interface-specific cellular responses.

Cao et al. develop porous poly-*L*-lactic acid (PLLA) microspheres to enhance dermal filler performance. Increased porosity improves surface area, cell interaction, and collagen production compared to solid particles, resulting in more uniform tissue integration. The porous structure also improves injectability, reduces aggregation, and lowers injection force. This work underscores how microscale architectural control can directly influence biological outcomes and clinical performance.

Pierre et al. introduce methacrylated decellularized heart matrix (DHMMA) as a photocrosslinkable, ECM-derived scaffold with tunable stiffness and degradation. By modulating functionalization and processing parameters, mechanical properties can be finely adjusted while retaining native biochemical cues. Microfabrication further enables topographical guidance of cell alignment, offering a versatile and biologically relevant platform for cardiac tissue engineering.

Dienemann et al. present a norbornene-functionalized gelatin (GelNB) hydrogel system for neural progenitor cell culture. Using a design-of-experiments approach, hydrogel stiffness and network architecture were precisely tuned within physiologically relevant ranges without compromising cell viability. The system supports cell clustering and reveals density-dependent hypoxia, highlighting the importance of both mechanical and metabolic microenvironments in neural tissue modeling.

De Vega et al. compare solvent-based and melt-processing methods for fabricating PCL–hydroxyapatite composite scaffolds for extrusion-based 3D printing. Solvent processing yields higher mechanical strength and faster degradation, whereas melt processing offers a cleaner, more scalable workflow. This comparison illustrates how fabrication routes influence scaffold properties and the trade-offs between performance and manufacturability.

Three **review articles** complement these original studies. Li et al. review natural products as radioprotective agents, emphasizing antioxidant, anti-inflammatory, and DNA-protective mechanisms while noting that most evidence remains preclinical. Li et al. examine the transition from platelet-rich formulations to extracellular vesicle–based therapies, highlighting improved stability and targeted bioactivity alongside challenges in standardization and regulation. Duan et al. review advances in 3D-printed bone scaffolds, focusing on material design, bioactive factor incorporation, and vascularization strategies, including the integration of vascularized tissue flaps for large-defect repair.

Collectively, these contributions demonstrate how advances in material chemistry, processing, and architecture converge to achieve increasingly precise control over biological function. Across these contributions, several unifying themes emerge. First, there is a clear shift from homogeneous materials toward **spatially and functionally heterogeneous systems**, as exemplified by multiphase scaffolds, porous microspheres, and ECM-derived hydrogels with tunable architectures. Second, **precise control over mechanical properties**, often enabled by chemical functionalization or systematic design approaches, remains central to directing cell behavior across tissues ranging from brain to myocardium to musculoskeletal interfaces. Third, the studies collectively highlight the importance of **microenvironmental regulation beyond mechanics**, including oxygen gradients, bioactive signaling, and structural anisotropy. Finally, advances in fabrication and processing, from photopolymerization to 3D printing, highlight how **processing-structure-property relationships** are increasingly leveraged to balance biological performance with manufacturability and scalability.

## 3 Outlook: future directions in polymer-based biomaterials

The contributions in this Research Topic underscore a broader shift in polymer-based biomaterials from passive structural supports toward **multifunctional, biologically instructive systems** capable of integrating mechanical, chemical, and microenvironmental cues. Across diverse applications that include musculoskeletal interfaces, cardiac and neural tissues, dermal regeneration, and bone repair, a central challenge remains the development of materials that can bridge scales, from molecular design to macroscopic function, within unified structure-property-performance frameworks (Zhang and Khademhosseini, 2017).

A key direction is the continued development of **heterogeneous and tissue-mimetic biomaterials** that recapitulate the compositional, structural, and mechanical complexity of native tissues (Li et al., 2017). As reflected across multiple studies in this collection, effective regeneration increasingly relies on scaffolds with spatially controlled gradients in stiffness, porosity, and bioactivity, enabling hard-to-soft transitions, anisotropy, and hierarchical organization. Emerging strategies (Li et al., 2025; Bubli et al., 2025) incorporating pro-angiogenic cues, extracellular vesicles, natural bioactive compounds, peptides, polysaccharides, and small molecules further support the development of materials that function as **dynamic, signal-regulating platforms** rather than static supports.

In parallel, there is increasing recognition that cellular response is governed not only by bulk stiffness but also by **viscoelasticity, degradation, transport behavior, fatigue, and local mechanical heterogeneity** (Chaudhuri et al., 2020). Future work must therefore move beyond static material descriptors to capture dynamic and spatially evolving cell-matrix interactions, particularly in tissues subject to complex mechanical loading or high metabolic demand.

Advances in fabrication, including electrospinning, cryogelation, photopolymerization, soft lithography, and additive manufacturing, further emphasize the importance of linking processing conditions to final scaffold structure and performance (Ballard et al., 2024). At the same time, translation will require addressing challenges in scalability, reproducibility, sterilization, storage stability, and regulatory readiness. Integrating design-of-experiments approaches, in situ characterization, and data-driven modeling will be critical for accelerating optimization and enabling predictive control over material systems (Tibbitt et al., 2009).

Overall, this Research Topic highlights that the future of polymer-based biomaterials lies in **integration across chemistry, structure, processing, and biological function**, with continued progress dependent on the development of adaptable, multifunctional systems that bridge fundamental materials design with practical considerations of manufacturability and clinical translation (Figure 1).
